# In vivo and in vitro studies on the sensitisation to a panel of allergens in a large rosacea allergic group of patients

**DOI:** 10.1186/2045-7022-1-S1-P99

**Published:** 2011-08-12

**Authors:** Ignacio Garcia Nuñez, Ana Aranda, Ana Belen Blazquez, Maria Jose Torres, Maria Luisa Galindo, Miguel Blanca, Maria Luisa Sanz

**Affiliations:** 1 Hospital Universitario Carlos Haya, Allergy Department, Malaga, Spain; 2 Clinica Universitaria de Navarra, Allergy Department, Pamplona, Spain

## Background

Allergy to peach and apple is a frequent problem in the Mediterranean area. Both fruits share allergens between them and with those from other plants and pollens. Component resolved diagnosis assays (CRD) enable to detect IgE antibodies to a wide panel of allergens. A detailed clinical evaluation plus CRD permit a precise analysis of sensitizations to many allergens. Our aim was to analyse sensitisation to fruit and pollen allergens by in vivo/in vitro methods in patients allergic to peach and/or apple.

## Methods

We included 107 patients. A detailed history, including questions related with response or tolerance to different fruits and plants, skin prick test (SPT) with a large panel of representative allergens in our area, and specific IgE antibodies using a CRD platform (ISAC, Phadia).

## Results

Sixty-six cases (61,68%) had symptoms with peel peach, 46(42,99%) with pulp peach and 21(19,62%) with apple. SPT with Pru p3 was positive in 53(49,53%), to Pru p1 in 9(8,41%) and to Mal d1 in 38(35,51%). CRD was positive to Pru p3 in 45(42,05%) and to Mal d1 in 6(5,6%). From the total group, 27(25,23%) tolerated peel peach, 47(43,92%) pulp peach and 76(71,02%) apple. Patients had skin test and CRD positive to allergens from fruits that they tolerated. Furthermore, different degree of clinical response and sensitization was obtained with all the other allergens evaluated.

## Conclusions

In vivo and in vitro evaluations with an extensive panel of allergens enable to make a precise diagnosis of allergic patients to fruits. However, discrepancies exist between clinical response and sensitization. Further studies are in progress for understanding these findings.

